# Different Heart Rate Patterns During Cardio-Pulmonary Exercise (CPX) Testing in Individuals With Type 1 Diabetes

**DOI:** 10.3389/fendo.2018.00585

**Published:** 2018-10-02

**Authors:** Othmar Moser, Gerhard Tschakert, Alexander Mueller, Werner Groeschl, Max L. Eckstein, Gerd Koehler, Richard M. Bracken, Thomas R. Pieber, Peter Hofmann

**Affiliations:** ^1^Exercise Physiology, Training Therapy & Training Research Group, Institute of Sports Sciences, University of Graz, Graz, Austria; ^2^Division of Diabetology & Metabolism, Department of Internal Medicine, Medical University of Graz, Graz, Austria; ^3^Diabetes Research Group, School of Medicine, Swansea University, Swansea, United Kingdom; ^4^Applied Sport, Technology, Exercise and Medicine Research Centre (A-STEM), College of Engineering, Swansea University, Swansea, United Kingdom; ^5^Sports Science Laboratory, Institute of Health and Tourism Management, FH JOANNEUM-University of Applied Sciences, Bad Gleichenberg, Austria

**Keywords:** exercise prescription, heart rate to performance curve, thresholds, type 1 diabetes, heart rate reserve

## Abstract

To investigate the heart rate during cardio-pulmonary exercise (CPX) testing in individuals with type 1 diabetes (T1D) compared to healthy (CON) individuals. Fourteen people (seven individuals with T1D and seven CON individuals) performed a CPX test until volitional exhaustion to determine the first and second lactate turn points (LTP_1_ and LTP_2_), ventilatory thresholds (VT_1_ and VT_2_), and the heart rate turn point. For these thresholds cardio-respiratory variables and percentages of maximum heart rate, heart rate reserve, maximum oxygen uptake and oxygen uptake reserve, and maximum power output were compared between groups. Additionally, the degree and direction of the deflection of the heart rate to performance curve (k_HR_) were compared between groups. Individuals with T1D had similar heart rate at LTP_1_ (mean difference) −11, [(95% confidence interval) −27 to 4 b.min^−1^], at VT_1_ (−12, −8 to 33 b.min^−1^) and at LTP_2_ (−7, −13 to 26 b.min^−1^), at VT_2_ (−7, −13 to 28 b.min^−1^), and at the heart rate turn point (−5, −14 to 24 b.min^−1^) (*p* = 0.22). Heart rate expressed as percentage of maximum heart rate at LTP_1_, VT_1_, LTP_2_, VT_2_ and the heart rate turn point as well as expressed as percentages of heart rate reserve at LTP_2_, VT_2_ and the heart rate turn point was lower in individuals with T1D (*p* < 0.05). k_HR_ was lower in T1D compared to CON individuals (0.11 ± 0.25 vs. 0.51 ± 0.32, *p* = 0.02). Our findings demonstrate that there are clear differences in the heart rate response during CPX testing in individuals with T1D compared to CON individuals. We suggest using submaximal markers to prescribe exercise intensity in people with T1D, as the heart rate at thresholds is influenced by k_HR_.

**Clinical Trial Identifier:** NCT02075567 (https://clinicaltrials.gov/ct2/show/NCT02075567).

## Introduction

Low amounts of structured exercise such as 3 × 30 min per week are associated with consistent improvements in health status in already healthy and physically active individuals (1). In people with both type 1 (T1D) and type 2 diabetes high doses of physical activity were found to be associated with a 40% and 29% lower risk of all-cause mortality and cardiovascular disease in comparison with inactivity (2).

Currently, standard exercise recommendations for individuals with T1D are similar to those for healthy individuals, namely 150 min or more of moderate to intense exercise and physical activity spread over at least three days per week, or a minimum of 75 min per week of vigorous-intensity exercise/physical activity (3, 4).

Intriguingly, the American Diabetes Association (ADA) recommends prescribing exercise intensity as percentages of maximum heart rate (5) even though there is an ongoing debate on how to prescribe exercise intensity (6, 7). It was shown that the variable time course of the heart rate to performance curve influences exercise prescription, translating to uncertainty to overestimate target training loads especially in the vigorous-intensity domain (8–11). Using percentages of maximum heart rate might be problematic as degree and direction of the deflection of the heart rate to performance curve (k_HR_) during CPX testing are altered in some healthy individuals (12) and might be further impaired by autonomic cardiac neuropathy in T1D individuals (13).

Furthermore, in T1D individuals with autonomic cardiac neuropathy, left ventricular function was found to be decreased in both systole and diastole, non-dipping was more prevalent, and pulse pressure was higher compared to patients without autonomic cardiac neuropathy (14). The heart rate to performance curve was shown to be related to myocardial function (15, 16). An increase in heart rate during CPX testing reflects the ability of the autonomic nervous system to respond to an increase in metabolic demands. In healthy individuals the increase in heart rate during CPX testing is mainly originated by a withdrawal of tonic vagal activity (17). As shown recently, individuals with T1D reveal a higher risk of impaired tonic vagal activity in comparison to their healthy counterparts (18). This impaired response of the autonomic nervous system might alter heart rate to performance curve, as found in our cohort.

As shown previously, different threshold concepts are suitable for healthy (6, 19) and T1D individuals (20, 21). The first threshold occurring during cardio-pulmonary exercise (CPX) testing [e.g., first lactate turn point (LTP_1_) or the first ventilatory threshold (VT_1_)] translates to the inability of the muscle to entirely oxidize produced lactate and therefore it is partly shifted into the blood stream and can be metabolized by other organs. At this first threshold there is still a metabolically balanced condition but on a systemic level (22). At the second threshold [e.g., second lactate turn point (LTP_2_) or the second ventilatory threshold (VT_2_)] produced lactate in the working muscles cannot be muscularly or systemically eliminated, resulting in a blood lactate accumulation (metabolically unbalanced condition, no lactate steady state) (6).

The aim of the study was to investigate the time course of the heart rate to performance curve during CPX testing in T1D individuals compared to matched healthy individuals (CON), and to prove its influence on percentages of maximum heart rate, percentages heart rate reserve, percentages maximum oxygen uptake, percentages oxygen uptake reserve and percentages maximum power output compared to standard markers of submaximal performance between groups. We determined as a primary outcome the heart rate to performance curve during CPX testing and its impact on target training heart rate determination in comparison of T1D individuals and matched healthy individuals (CON) and as secondary outcomes the functional capacity, cardiorespiratory and metabolic markers in comparison of groups during CPX testing. We hypothesized that people with T1D show an altered heart rate to performance curve during CPX testing and this impacts target training heart rate determination.

## Methods

### Consent procedures

This study was carried out in accordance with the recommendations of Good Clinical Practice (GCP), “Bundesamt fuer Sicherheit im Gesundheitswesen” (BASG Austria). The protocol was approved by the ethics committee of the Medical University of Graz, AT (26-069 ex 13/14). All subjects gave written informed consent in accordance with the Declaration of Helsinki. CON individuals were matched from routine diagnostics (approved by the Ministry of Science).

### Eligibility criteria and assessment

To be eligible for the study, T1D individuals had to be male, diagnosed with T1D for at least 12 months, aged between 18 and 35 years (both inclusive), glycated hemoglobin (HbA_1c_) ≤ 8% (64 mmol.mol^−1^), fasting c-peptide negative, treated with multiple daily insulin injections, no long-term complications and no other physical and/or mental diseases, which might influence the study results. In this primary investigation we recruited male individuals as the female menstrual cycle may influence the energy supply during CPX testing (23). Participants were excluded if they had a history of any disease that might confound the results of the trial, use of drugs, which may interfere with the interpretation of the trial's results or known to be clinically relevant in interfering with insulin action, glucose utilization or recovery from hypoglycemia, current addiction to alcohol or any controlled substance abuse. Furthermore, participants were excluded if they had known or suspected allergy to trial products or related products, mental incapacity, unwillingness, language barriers precluding adequate understanding, and any condition that the study physician feels would interfere with the trial participation. Testing day exclusion criteria were: hypoglycemia 48 h prior to testing, illness on or before the testing day, low glucose levels immediately before testing (≤ 4.4 mmol.l^−1^) or alcohol consumption 24 h before. All data were assessed and documented in a standardized Case Report Form. CON individuals were selected with respect to T1D individuals' characteristics (matched for sex, age, and maximum power output) from routine diagnostics data at the entry to the physical education studies. CON individuals were instructed to avoid intense or long-lasting exercise 24 h prior to the investigation and to avoid alcohol within 24 h pre-testing. Written informed consent was obtained from all participants.

### Participants characteristics

T1D individuals' characteristics were: age 24 ± 5 years (min–max; 19–32), BMI 23.9 ± 2.5 kg.m^−2^ (20–28), HbA_1c_ 7.4 ± 0.6% (6.5–8) (57 ± 6.3 mmol.mol^−1^) (47–64), c-peptide 0.13 ± 0.19 nmol.l^−1^ (0–0.4) and total daily insulin dose with insulin Degludec 41 ± 16 U (27–69). Four participants used bolus insulin Aspart (Novo Rapid, Novo Nordisk, Denmark) and three participants used bolus insulin Lispro (Humalog/ Lilly, USA). Before switching to insulin Degludec, two patients used insulin Detemir (Levemir/ Novo Nordisk, Denmark) and five patients used insulin Glargine (Lantus/ Sanofi- Aventis, France) as basal therapy. T1D participants were free of comorbidities and co-medications other than insulin. CON individuals were aged 23 ± 4 years (19–30), had a BMI of 23.4 ± 1.8 kg.m^2^ (21–26) and were healthy without taking any medications.

### Study procedures

In this experimental design, T1D individuals were adjusted to the same therapy with insulin Degludec (®Tresiba/Novo Nordisk, Dagsvaerd, Denmark) as part of a basal-bolus routine. After a run-in period of 4 weeks with insulin Degludec, a CPX test was performed. Insulin Degludec was used to achieve homogeneity for the basal insulin therapy. We have chosen insulin Degludec since its pharmacodynamic profile is flat and stable, demonstrated by an even distribution of glucose-lowering effects across the 24-h period (24).

### Cardio-pulmonary exercise (CPX) testing

Fourteen participants performed a maximum CPX test on a cycle ergometer (Monark Ergomedic 839E, Monark, Sweden) at the Sports Science Institute, University of Graz, Austria (25). Participants were permitted to cycle at a cadence of 70–90 rpm. At the beginning of the CPX testing, participants sat on the cycle ergometer for 3 min without pedaling (0 W). Then, participants started to cycle for 3 min with a workload of 40 W. Subsequently, the workload was increased by 20 W every minute until volitional exhaustion, followed by 3 min of active recovery at 40 W and 3 min of passive recovery (0 W). LTP_1_ and LTP_2_ (12) as well as VT_1_ and VT_2_ were determined from the CPX test by means of a computer-based linear regression break point analysis (6). LTP_1_ was defined as the first increase in capillary blood lactate concentration above baseline values and LTP_2_ was defined as the second abrupt increase between LTP_1_ and the maximum power output. VT_1_ was defined as the first increase in ventilation (VE) accompanied by an increase in VE/VO_2_ without an increase in VE/VCO_2_. VT_2_ was defined as the second abrupt increase in VE accompanied by an increase in both VE/VO_2_ and VE/VCO_2_. Additionally, the heart rate turn point was defined as the point of intersection of two regression lines in the heart rate to performance curve between LTP_1_ and the maximum power output with minimal standard deviation of the two straight lines. The degree and direction of the deflection of the heart rate to performance curve (k_HR_) was calculated by a second-degree polynomial function between LTP_1_ and the maximum power output (12, 21).

## Measurements

Pulmonary gas-exchange variables were measured continuously during CPX testing via breath-by-breath measurement and 5 s average (ZAN 600, ZAN, Germany). Heart rate was measured continuously via chest belt telemetry and 5 s average (PE 4000, Polar Electro, Finland). A 12-lead electrocardiogram and blood pressure measurements (every 2 min) were obtained during CPX testing for cardiac monitoring. Blood lactate and blood glucose (for safety in T1D group) concentrations were determined by taking capillary blood samples from the earlobe at the end of the rest and warm-up periods, at the end of each workload step (every minute), and at the end of active and passive recovery. Blood lactate and blood glucose were analyzed by means of an enzymatic-amperometric method (®Biosen S-line, EKF Diagnostics, Germany).

### Bolus insulin dose reduction

After overnight fasting, T1D individuals received a standardized meal on the day of the CPX test (®Fortimel Extra, Nutricia GmbH, Germany), which was calculated from the average pre-investigational amount of consumed carbohydrates during the last 4 weeks prior to the start of the study. The standardized meal and the reduced bolus insulin dose were administered exactly 4 h before the cycle ergometer exercise tests. The bolus insulin dose was reduced by 40% of the regular dose for the CPX testing. Both groups were informed to ingest carbohydrate enriched meals at least the day prior CPX testing to ensure adequate tissue glycogen stores.

### Statistical analyses

All data were tested with Shapiro-Wilk normality test and were found to be normally distributed. Descriptive statistics included mean and standard deviation for participant's anthropometric data, performance characteristics, and diabetes specific data. Anthropometric data and k_HR_ were compared between groups by paired students' *t*-test. Differences in comparison of groups for thresholds LTP_1_, VT_1_, LTP_2_, VT_2_, and heart rate turn point for relative and absolute power output, heart rate, oxygen consumption and lactate concentration were analyzed by a two-way ANOVA (group and threshold), with Bonferroni *post-hoc* testing. All statistics were performed with a standard software package ®Prism Software version 5.0 (GraphPad, USA) and SPSS 22.0 software (SPSS Inc., USA).

## Results

*Post-hoc* power analysis was performed for the primary outcome percentages of heart rate comparing markers of lowest significant difference between groups (VT_1_) and revealed a *post hoc* power 1-β = 0.99. There were no episodes of hypoglycemia noted during the CPX testing in the T1D group. T1D individuals maintained a blood glucose steady state during CPX testing showing no significant difference between the start (10.58 ± 3.42 mmol.l^−1^) and the end concentrations (10.24 ± 3.48 mmol.l^−1^) (*p* = 0.86).

Cardio-respiratory and metabolic markers at LTP_1_ and VT_1_ [T1D: *p* = 0.98 (heart rate), *p* = 0.99 (oxygen uptake), *p* = 0.92 (lactate concentration); CON: *p* = 0.96 (heart rate), *p* = 0.60 (oxygen uptake), *p* = 0.96 (lactate concentration)] as well as LTP_2_, VT_2_ and heart rate turn point [T1D: *p* = 0.93 (heart rate), *p* = 0.99 (oxygen uptake), *p* = 0.58 (lactate concentration); CON: *p* = 0.37 (heart rate), *p* = 0.97 (oxygen uptake), *p* = 0.76 (lactate concentration)] were not significantly different within both groups.

No significant differences were found for power output, heart rate, oxygen uptake and lactate concentration at LTP_1_, LTP_2_, heart rate turn point, and maximum power output between T1D and CON individuals, except for lactate concentration at LTP_1_ (Table 1).

**Table 1:** Comparison of anthropometric data, performance data and physiological data during CPX testing.

	**T1D (*n* = 7)**	**CON (*n* = 7)**	***p*-value**
Age (years)	24.5 ± 5.3	23.4 ± 4.1	0.47
BMI (kg/m^2^)	23.9 ± 2.5	23.4 ± 1.8	0.70
HbA_1c_ (%)	7.4 ± 0.6	–	–
HbA_1c_ (mmol.mol^−1^)	57 ± 6.3	–	–
Diabetes duration (years)	16.9 ± 8.1	–	–
P_LTP1_ (Watt)	83 ± 18	92 ± 22	0.41
P_VT1_ (Watt)	82 ± 8	103 ± 12	0.17
P_LTP2_ (Watt)	192 ± 33	197 ± 30	0.74
P_VT2_ (Watt)	193 ± 14	198 ± 15	0.80
P_HRTP_ (Watt)	198 ± 40	197 ± 30	0.93
P_max_ (Watt)	284 ± 43	296 ± 37	0.58
HR_LTP1_ (b.min^−1^)	116 ± 14	127 ± 12	0.13
HR_VT1_ (b.min^−1^)	116 ± 5	128 ± 5	0.14
HR_LTP2_ (b.min^−1^)	159 ± 11	165 ± 10	0.28
HR_VT2_ (b.min^−1^)	159 ± 4	166 ± 5	0.17
HR_HRTP_ (b.min^−1^)	160 ± 10	165 ± 10	0.38
HR_max_ (b.min^−1^)	192 ± 4	187 ± 11	0.30
VO_2LTP1_ (ml.kg^−1^.min^−1^)	19.88 ± 14	20.53 ± 2.73	0.80
VO_2VT1_ (ml.kg^−1^.min^−1^)	19.89 ± 2.75	19.73 ± 1.11	0.95
VO_2LTP2_ (ml.kg^−1^.min^−1^)	38.20 ± 9.05	38.26 ± 4.21	0.98
VO_2VT2_ (ml.kg^−1^.min^−1^)	38.67 ± 3.50	38.07 ± 2.02	0.88
VO_2HRTP_ (ml.kg^−1^.min^−1^)	39.82 ± 10.93	38.61 ± 4.58	0.79
VO_2max_ (ml.kg^−1^.min^−1^)	52.49 ± 6.56	52.39 ± 8.57	0.98
LA_LTP1_ (mmol.l^−1^)	0.91 ± 0.24	1.50 ± 0.58	0.03^*^
LA_VT1_ (mmol.l^−1^)	0.90 ± 0.11	1.48 ± 0.24	0.05
LA_LTP2_ (mmol.l^−1^)	3.68 ± 0.53	4.10 ± 0.50	0.16
LA_VT2_ (mmol.l^−1^)	3.75 ± 0.23	4.18 ± 0.21	0.20
LA_HRTP_ (mmol.l^−1^)	3.98 ± 0.49	3.88 ± 1.12	0.83
LA_max_ (mmol.l^−1^)	12.37 ± 1.25	13.10 ± 0.79	0.21

*T1D: individuals with type 1 diabetes, CON: healthy individuals, LTP1, first lactate turn point; LTP2, second lactate turn point; HRTP, heart rate turn point; max, maximum output; P, power output; HR, heart rate; VO2, oxygen uptake; LA, lactate concentration; Values are given as mean ± SD*.

* *represents significant difference*.

**Figure 1:**
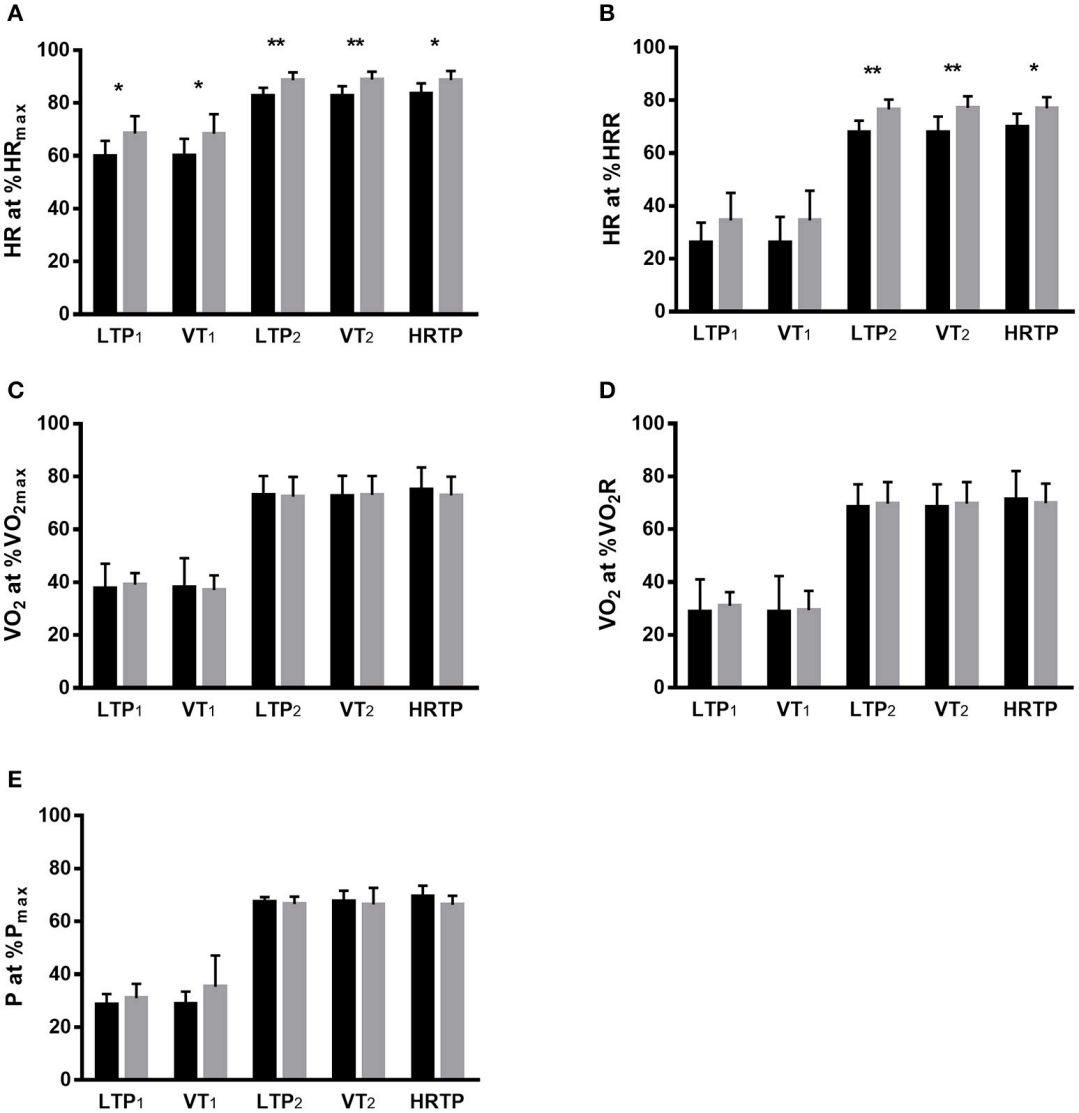
Comparison of CPX-testing derived markers between both groups for HR at %HR_max_ **(A)**, HR at %HRR **(B)**, VO_2_ at %VO_2max_ **(C)**, VO_2_ at %VO_2_R **(D)** and P at %P_max_ **(E)**. %HR_max_, percentage of maximum heart rate; %HRR, percentage of heart rate reserve; %VO_2max_, percentage of maximum oxygen uptake; %VO_2_R, percentage of oxygen uptake reserve; %P_max_, percentage of maximum power output; LTP_1_, first lactate turn point; LTP_2_, second lactate turn point; HRTP, heart rate turn point; VT_1_, first ventilatory threshold; VT_2_, second ventilatory threshold. Values are given as mean ± SD. Stars represent significant difference, *p < 0.05, ^**^p < 0.01. Black bar, individuals with type 1 diabetes; grey bar, healthy individuals.

**Figure 2:**
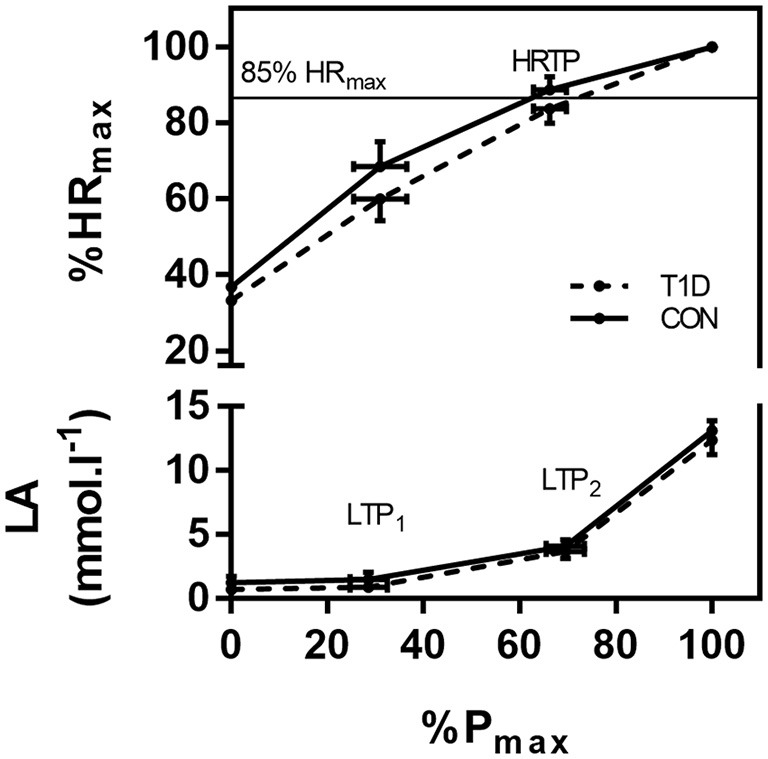
Comparison of the relative heart rate performance curves and lactate curves between both groups. The same relative intensity of 85% HR_max_, which is a usual upper limit for moderate intensity, resulted in different workloads related to the LTP_2_. %HR_max_, percentage of maximum heart rate; LA, lactate concentration; %P_max_, percentage of maximum power output; LTP_1_, first lactate turn point; LTP_2_, second lactate turn point; HRTP, heart rate turn point. Values are given as mean ± SD.

Significantly lower values were found for heart rate at percentages of maximum heart rate at LTP_1_, VT_1_, LTP_2_, VT_2_, and heart rate turn point as well as for heart rate at percentages of heart rate reserve at LTP_2_, VT_2_, and heart rate turn point in T1D individuals (*p* < 0.05). No significant differences were found for the oxygen uptake at percentages of maximum oxygen uptake (*p* = 0.88), oxygen uptake at percentages of oxygen uptake reserve (*p* = 0.71) and heart rate at percentages of heart rate reserve (only for LTP_1_ and VT_1_, *p* = 0.11) as well as percentages of maximum power output %P (*p* = 0.64) at LTP_1_, VT_1_, LTP_2_, VT_2_, and heart rate turn point when comparing T1D and CON individuals (Figure 1).

k_HR_ was significantly lower in T1D individuals compared to CON individuals (0.11 ± 0.25 vs. 0.51 ± 0.32, *p* = 0.02). Figure 2 shows the time course of the heart rate to performance curve for both groups indicating the differences especially important for the upper limit for vigorous intensity exercise. Usually, maximum heart rate derived calculations of the target training heart rate overestimate the true limits, as this can be seen via lactate turn points (Figure 2). The usual upper limit of 85% of the maximum heart rate was clearly above the exercise intensity of LTP_2_ for T1D individuals and clearly below LTP_2_ for CON individuals. Eighty-five percent of the maximum heart rate resulted in significant differences comparing T1D vs. CON for oxygen uptake at percentages of maximum oxygen uptake [mean difference (MD) 11.57, confidence interval (CI) 0.15 to 22.99 %, *p* = 0.04], oxygen uptake at percentages of oxygen uptake reserve (12.86, 1.43 to 24.28, *p* = 0.02), and power output at percentages of maximum power output (12.57, 1.15 to 23.99 %, *p* = 0.02), but not for heart rate at percentages of heart rate reserve (3.71, −7.70 to 15.13 %, *p* = 0.87).

## Discussion

The aim of this study was to investigate the alterations in the heart rate during CPX testing in T1D individuals compared to matched CON individuals. k_HR_ was significantly different between T1D individuals and CON individuals. T1D individuals showed a lower heart rate of percentages of the maximum heart rate at the second turn points (LTP_2_, VT_2_, heart rate turn point) and a significantly lower k_HR_ (Figure 2), which significantly influenced prescription of the target heart rate. Applying percentages of maximum heart rate derived markers overestimated T1D individuals' target training heart rate which is important especially for the upper limits for vigorous intensity exercise. All threshold values within groups were not significantly different (LTP_1_ vs. VT_1_; LTP_2_ vs. VT_2_ vs. heart rate turn point) indicating the objectivity and the validity of these markers which may be used interchangeably.

Different methods to prescribe exercise intensity for physical training show different advantages and disadvantages. As shown by our data, using percentages of maximum values [e.g., maximum heart rate or oxygen uptake (6)] is not sufficient to prescribe exercise intensity individually and might be impaired in patients with cardio-respiratory diseases. Submaximal threshold concepts (e.g., lactate-, ventilatory- and heart rate-derived) are a valid approach due to their theoretical foundation and its internal consistency. However, it must be stated that especially rater-depending software programs might be influenced by subjectivity that can alter exercise intensity prescription. Although oxygen uptake at percentages of maximum oxygen uptake, oxygen uptake at percentages of oxygen uptake reserve and heart rate at percentages of heart rate reserve (only for LTP_1_ and VT_1_) were not significantly different at all submaximal markers, heart rate at percentages of maximum heart rate at LTP_1_, VT_1_ and LTP_2_, VT_2_ and heart rate turn point as well as percentages of heart rate reserve at LTP_2_, VT_2_ and heart rate turn point were significantly lower in T1D individuals compared to CON individuals. It was previously shown that these differences were also found in young healthy and trained subjects (26) with about 15% of non-regular heart rate curves. However, in our study 62.5% of T1D individuals showed an atypical heart rate response.

In several studies, it was shown that k_HR_ was associated with left ventricular ejection fraction (12, 16, 27) and β_1_-adrenoceptor sensitivity (28). In particular chronically elevated HbA_1c_ levels and concomitantly elevated catecholamine levels (29) or inflammation (30) may impair β_1_-adrenoreceptor sensitivity. This might change the degree and direction of the deflection of the heart rate to performance curve as shown earlier by Wonisch et al. (10). Apart from the effects of chronic hyperglycemia, hypoglycemic episodes might further promote impaired cardiovascular function (31). T1D individuals experience on average two episodes of symptomatic hypoglycemia per week and at least one severe episode per year (32). Additionally, the incidence of severe hypoglycemia increases drastically with length of diabetes. Both hyperglycemia and recurrent hypoglycemia might be perpetrators for an impaired cardiac response during CPX testing in individuals with T1D. Overall, our data show a systematic overestimation of target heart rate in T1D individuals when applying percentages of maximum heart rate compared to objective individual markers of submaximal performance such as LTP_1_, VT_1_, LTP_2_, VT_2_, and heart rate turn point.

With respect to the findings in our study we consequently recommend using percentages of submaximal markers [e.g., the first and the second turn points for lactate (LTP_1_ and LTP_2_) or ventilatory variables (VT_1_, VT_2_)] for the exercise intensity prescription in T1D which were shown to be representative for constant and intermittent type exercise of various intensities (6, 20, 25). As shown in Figure 2, individuals with T1D might be exercising at a too high exercise intensity if exercise is prescribed by percentages of maximum heart rate. From a clinical point of view exercise intensity should be prescribed exactly to avoid both, unexpected hypoglycemia and to induce training effects. Since insulin needs to be reduced with light to moderate exercise intensity to avoid peri-exercise hypoglycemia, glucose levels remain relatively stable during exercise with vigorous intensity (33) and rise immediately after intense exercise is performed (34). If exercise intensity and the insulin dose reduction do not match accordingly, the risk of glycemic impairments rises (35) which could lead to severe health complications, including death (36). On the other hand, if the exercise intensity is close or below the LTP_1_, sub-optimal training effects on cardio-respiratory fitness may be expected, which reduces the possibility of attaining the accompanied health benefits.

To the best of our knowledge, this is the first study investigating the differences in cardio-pulmonary responses of the heart rate, heart rate reserve, oxygen uptake, and oxygen uptake reserve as well as maximum power output in relation to objective submaximal markers LTP_1_, VT_1_, LTP_2_, VT_2_, and heart rate turn point during CPX testing in T1D individuals and age, gender- and maximum power output- matched CON individuals. This study is limited by the small number of participants and that only males were included, which makes a direct transfer of the results to the general population of individuals with T1D difficult. Additionally, CON individuals were not provided with the same meal prior to the CPX testing as the T1D individuals. Further large-scale studies are needed investigating the heart rate to performance curve, which is obviously altered in this population. However, *post-hoc* power analysis confirmed the relevance of this study (power 1-β = 0.99).

In conclusion, our findings demonstrate that there are clear differences in heart rate responses during CPX testing in individuals with T1D compared to CON individuals. We postulate that T1D individuals displayed an altered k_HR_ and lowered percentages of maximum heart rate / percentages of heart rate reserve at submaximal markers. We recommend additional studies to analyze e.g., HbA_1c_ levels in relation to the heart rate to performance curve in a larger group of individuals with T1D.

## Consent of publication

Both groups gave written signed informed consent for publication.

## Availability of data and material

Data will be made available on demand by the corresponding authors via email contact.

## Author contributions

All authors confirm that they meet the International Committee of Medical Journal Editors (ICMJE) uniform requirements for authorship. OM participated in the conceiving and designing the study, analyzing the data, and writing the manuscript. GT, AM, WG, and GK conceived the study and conducted the measurements. ME and RB conceived the study, analyzed data, drafted and revised the manuscript. TP and PH designed the study, supervised the measurements, and writing process. All authors have approved the final version of the manuscript to be published.

### Conflict of interest statement

OM has received lecture fees from Medtronic, travel and research fees form Novo Nordisk A/S, Novo Nordisk Austria and Dexcom Inc. and received a grant from Ser Cymru II COFUND fellowship/European Union. ME has received a KESS2/European Social Fund scholarship. RB has received educational grants from Novo Nordisk, Eli Lilly, Sanofi, Boehringer Ingelheim, and Beneo. GK has received lecture fees and has participated in advisory panels from Novo Nordisk, Bohringer Ingelheim, Novartis, MSD, and Eli Lilly. TP has participated in advisory panels and acted as a consultant for Novo Nordisk. The remaining authors declare that the research was conducted in the absence of any commercial or financial relationships that could be construed as a potential conflict of interest.

## Abbreviations

T1D: Type 1 diabetes/individuals with Type 1 diabetes
HbA_1c_: Glycated hemoglobin
BMI: Body mass index
ADA: American Diabetes Association
%HR_max_: Percentages of maximum heart rate
HR: Heart rate
k_HR_: Degree and direction of the deflection of the heart rate to performance curve
CPX: Cardio-pulmonary exercise testing
CON: Healthy individuals
%HRR: Percentages of heart rate reserve
%VO_2max_: Percentages of maximum oxygen uptake
%VO_2_R: Percentages of oxygen uptake reserve
%P_max_: Percentages of maximum power output
GCP: Good clinical practice
DoH: Declaration of Helsinki
MDII: Multiple daily insulin injections
CRF: Case Report Form
P_max_: Maximum power output
LTP_1_: First lactate turn point
LTP_2_: Second lactate turn point
VT_1_: First ventilatory threshold
VT_2_: Second ventilatory threshold
VE: Ventilation
VE/VO_2_: Ventilation of oxygen
VE/VCO_2_: Ventilation of carbon dioxide
HRTP: Heart rate turn point
MD: Mean difference
CI: confidence interval.
